# Thyroid hemiagenesis

**DOI:** 10.1093/qjmed/hcad054

**Published:** 2023-04-06

**Authors:** E Kaba, M Solak

**Affiliations:** Department of Radiology, Faculty of Medicine, Recep Tayyip Erdoğan University, Rize, Turkey; Department of Radiology, Faculty of Medicine, Recep Tayyip Erdoğan University, Rize, Turkey

**Figure 1.**
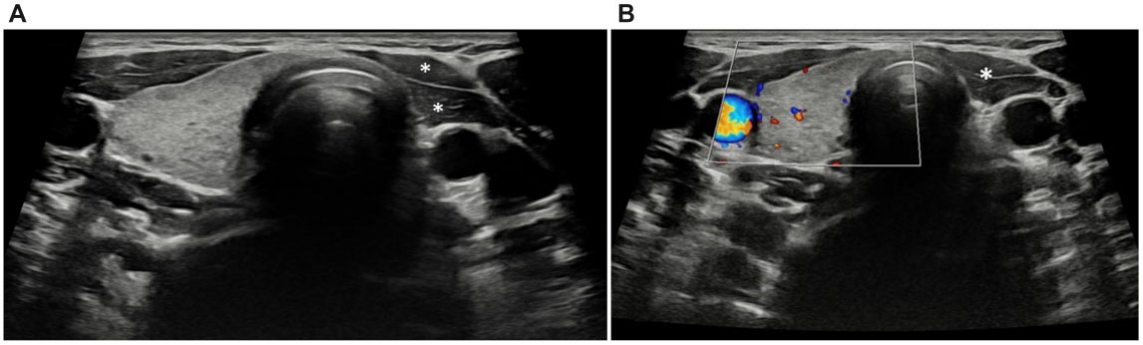
(**A**) Strap muscles are observed instead of the left thyroid lobe (*). (**B**) Vascularity and echogenicity of the right thyroid lobe are normal.

A 17-year-old female patient presented with complaints of fatigue and hair loss. A blood test revealed Thyroid Stimulating Hormone (TSH): 5.23 µU/ml and T4: 1.21 µU/ml. Subclinical hypothyroidism was considered and thyroid ultrasonography was performed. The left lobe of the thyroid and the left half of the isthmus were not observed. Right lobe parenchyma was normal. Doppler examination showed a normal pattern (Figure 1). There was no history of any surgery. Therefore, the diagnosis was made as thyroid hemiagenesis.

Thyroid hemiagenesis is the congenital absence of one lobe of the thyroid gland. It is extremely rare. The prevalence rates vary between 0.05% and 0.5% and it is a congenital variation that is more common in females.^1^ Left lobe deficiency is frequently seen. Patients who are mostly asymptomatic are usually detected at a late age on incidental thyroid ultrasonography examination. In these patients, the contralateral thyroid gland may be normal, or compensatory hypertrophy or hyperplasia may be seen. There is an increased risk of pathology in the normal lobe.^2^

In the sonographic examination of these patients, it is necessary to evaluate for the possible presence of ectopic thyroid tissue in the neck.^3^
